# *Cryptosporidium parvum* Subverts Antimicrobial Activity of CRAMP by Reducing Its Expression in Neonatal Mice

**DOI:** 10.3390/microorganisms8111635

**Published:** 2020-10-23

**Authors:** William Guesdon, Tiffany Pezier, Sandrine Menard, Alessandra Nicolosi, Yves Le Vern, Anne Silvestre, Julien Diana, Fabrice Laurent, Sonia Lacroix-Lamandé

**Affiliations:** 1 INRAE, Université de Tours, ISP, F-37380 Nouzilly, France; wguesdon@gmail.com (W.G.); Tiffany.Pezier@inrae.fr (T.P.); alessandra.f.nicolosi@gmail.com (A.N.); yves.levern@inrae.fr (Y.L.V.); anne.silvestre@inrae.fr (A.S.); fabrice.laurent@inrae.fr (F.L.); 2 INRAE Centre de Toulouse, UMR 1331 INRA/INP/UPS TOXALIM, F-31027 Toulouse, France; sandrine.menard@inrae.fr; 3 Inserm Unité 1151 Institut Necker-Enfants Malades, Inserm/CNRS/Université Paris Descartes, F-75730 Paris, France; julien.diana@inserm.fr

**Keywords:** *Cryptosporidium parvum*, CRAMP, antimicrobial activity, neonatal mice

## Abstract

*Cryptosporidium parvum* causes diarrhea in infants under 5 years, in immunosuppressed individuals or in young ruminants. This parasite infects the apical side of ileal epithelial cells where it develops itself and induces inflammation. Antimicrobial peptides (AMPs) are part of the innate immune response, playing a major role in the control of the acute phase of *C. parvum* infection in neonates. Intestinal AMP production in neonates is characterized by high expressions of Cathelicidin Related Antimicrobial Peptide (CRAMP), the unique cathelicidin in mice known to fight bacterial infections. In this study, we investigated the role of CRAMP during cryptosporidiosis in neonates. We demonstrated that sporozoites are sensitive to CRAMP antimicrobial activity. However, during *C. parvum* infection the intestinal expression of CRAMP was significantly and selectively reduced, while other AMPs were upregulated. Moreover, despite high CRAMP expression in the intestine of neonates at homeostasis, the depletion of CRAMP did not worsen *C. parvum* infection. This result might be explained by the rapid downregulation of CRAMP induced by infection. However, the exogenous administration of CRAMP dampened the parasite burden in neonates. Taken together these results suggest that *C. parvum* impairs the production of CRAMP to subvert the host response, and highlight exogenous cathelicidin supplements as a potential treatment strategy.

## 1. Introduction

Cryptosporidiosis is a zoonosis associated with foodborne and waterborne outbreaks. The protozoan parasite *Cryptosporidium* is one of the leading causes of infectious diarrhea in children and young ruminants. In Africa and Asia, it is now the second cause of diarrhea in infants under five after rotaviruses, and it increases mortality rate, impairs child development and favors developmental deficits [1]. In livestock species, diarrhea caused by *Cryptosporidium* is prevalent throughout the world and is responsible for important economics losses for farmers [2].

This parasite infects specifically small intestinal epithelial cells (IECs). We previously demonstrated that the *Cryptosporidium parvum* (*C. parvum*) infection of small intestinal epithelial cells induces apoptosis [3,4] and the secretion of a broad range of chemokines [5,6] and several pro-inflammatory cytokines, allowing inflammatory cell recruitment to the site of infection [6]. We demonstrated that this interplay between IECs and dendritic cells is essential for the innate control of the acute phase of this infection [7]. Antimicrobial peptides (AMPs) are considered as an essential part of the innate immune response. Some of them, such as β-defensin, human cathelicidin LL-37 and CCL20, can efficiently kill *C. parvum* sporozoites in vitro [8,9,10].

Cathelicidins are a family of antimicrobial peptides playing a critical role in the innate immune defense against invasive bacterial infections [11]. Cathelicidins exert antibacterial activities against both Gram-positive and Gram-negative bacteria via electrostatic interactions with the bacterial cell membrane [12,13,14]. Members of the cathelicidin family are characterized by an N-terminal highly conserved region (cathelin domain) and a highly variable C-term cathelicidin peptide domain. This C-term domain exerts antimicrobial activity. They can be produced by neutrophils, macrophages and epithelial cells. In addition to bactericidal activity, cathelicidins exert a number of immunomodulatory effects, including LPS binding and neutralization, the chemotaxis of immune cells, and stimulating the release of inflammatory cytokines [15,16]. Only one cathelicidin is described in humans and mice, Cathelicidin Related Antimicrobial Peptide (CRAMP) being the mouse homologue to the human cathelicidin LL-37/hCAP-18, while multiple cathelicidin genes have been found in cattle and sheep [17]. In the newborn intestine, this antimicrobial peptide is strongly produced in the small intestine in the first two weeks after birth in mice, and then gradually disappears [18]. This age-dependent epithelial expression contributes to the protection of neonates from enteric infections, such as the enteric pathogen *Listeria monocytogenes* [18].

As such, the present study aimed to investigate the role of CRAMP during cryptosporidiosis, an intestinal infection occurring during the neonatal period in WT mice. Many studies have shown that AMPs, such as cathelicidins, can alter *C. parvum* sporozoite viability in vitro [8,9,19], suggesting that they may play an important role in *C. parvum* infection. Therefore, in the present investigation, we explored the possible effects of the murine cathelicidin CRAMP both in vitro and in vivo in the neonatal mouse model. Overall, this study demonstrates that CRAMP not only directly inhibits *C. parvum* viability, but also reduces *C. parvum* infection when administered to neonatal mice. This work thus provides new evidence for the therapeutic value of cathelicidin against *C. parvum* infection.

## 2. Materials and Methods

### 2.1. Parasite

Oocysts of the *C. parvum* CpINRA isolate were initially purified as previously described and used within 2 months [20]. For the antimicrobial assay, in vitro sporozoite excystation was performed by incubating oocysts with taurocholic acid (0.75%) and porcine trypsin (0.25%) diluted in phosphate-buffered saline (PBS) for 30 min at 37 °C. Sporozoites were purified by passage through a 5 µm seringe-polycarbonate filter.

### 2.2. Mouse Models and Ethic Statements

C57BL/6 IFNγ^−/−^, CD11c-DTR, CRAMP^−/−^ mice and C57BL6/J Wild-Type (WT) mice were raised and maintained in PFIE facilities (INRAE-Tours) and germ-free mice (GF) in the Anaxem platform facilities (INRAE-Jouy en Josas), all under specific pathogen-free conditions. For all experiments, three-day-old neonates were orally infected with 5 × 10^5^ oocysts. The level of infection in neonates was assessed by counting oocysts in the intestinal content, as already described [20]. All experimental protocols were conducted in compliance with French legislation (Décret: 2001-464 29/05/01) and EEC regulations (86/609/CEE) governing the care and use of laboratory animals, after validation by the local ethics committee for animal experimentation (Comité d’Ethique pour l’Expérimentation Animale Val de Loire (CEEA VdL)): APAFIS#284.

### 2.3. mICcl2 Cell Line Culture and Viability

The murine intestinal cell line mICcl2 was kindly provided by A. Vandewalle (Inserm, U773, Centre de Recherche Biomédicale Bichat-Beaujon, Paris, France) and was maintained in a growth medium as described [21]. To assess their viability, epithelial cells were incubated with MTT (Sigma-Aldrich, St Louis, MO, USA) for 4 h and OD was measured at 570 nm.

### 2.4. Test of Sporozoite Viability

Sporozoites of *C. parvum* were incubated for 3 h at 37 °C with human cathelicidin (LL-37, Phoenix Pharmaceuticals, Belmont, CA, USA) or mouse CRAMP (Bachem, Bubenforf, Switzerland) while control sporozoites were heat-killed for 20 s at 100 °C in phosphate buffer containing 50 mM NaCl. The parasite viability was assessed with a MoFlo cell sorter Astrios (Dako, Denmark) on 30,000 sporozoites analyzed after incubation with Carboxyfluorescein diacetate succinimidyl ester (CFDA-SE) (Molecular Probes, Eugene, OR, USA) (8 µg/mL) and propidium iodide (Sigma-Aldrich, St Louis, MO, USA) (5 µg/mL) at 21 °C for 5 min.

### 2.5. Isolation of Intestinal Epithelial Cells (IEC)

The purification of IEC was performed as described previously [10]. Briefly, after opening the small intestine longitudinally, several washes with HBSS without Ca^2+^ and Mg^2+^ (were performed. Then, the small intestine was transferred into a flask with ice-cold Matrisperse cell recovery solution (Corning Life Sciences, Tewksbury, MA, USA). After 4 h, the intestine was rigorously shaken to separate the epithelium. The mixture was filtered at 100 µm, then at 60 µm, and the epithelium was recovered on the 60 µm filter. Purified cells from 3 neonatal mice were incubated with Trizol (Life Technologies Corporation, Carlsbad, CA, USA) and frozen at −80 °C until processing.

### 2.6. RNA Extraction and qRT-PCR

Total RNAs were extracted using Trizol reagent solution. A Reverse Transcriptase (RT) reaction was performed with 1 µg of total RNA using oligo(dT)15 primers or random hexamers with M-MLV reverse transcriptase (Promega, Madison, WI, USA) in 20 µL final volume. Quantitative RT-PCR was performed with 2 µL of reverse transcription reaction (previously diluted 1:10) in a 15 µL total reaction with Sso advanced SYBR Green Supermix (Biorad, Hercules, CA, USA) on a CFX96 (Biorad laboratories Inc.) [10]. PCR relies on two thermal cycling steps to amplify a target DNA sequence, and these are run for 40 cycles. The expression levels were calculated using the formula 2(−^ΔCt^), where ΔCt for each sample = Ct gene−Ct average housekeeping. TATA-Box Binding Protein (TBP) and Peptidylprolyl isomerase A (PPia) served as housekeeping genes. Primer sequences of genes are presented in Table 1 [7,10]. They were designed in order to avoid genomic amplification by either flanking a large intron or spanning an exon–exon junction.

### 2.7. Lamina propria Cell Preparation and Flow Cytometry

Immune cells from *lamina propria* were purified as previously described [7]. They were incubated with anti-CD16/CD32 (2.4G2) antibody in PBS, 1% FCS, 2 mM EDTA and various antibodies, including anti-CD3ε FITC (17A2), anti-CD11c APC (N418), anti-IA/IE FITC (2G9) and anti-F4/80 APC (BM8) from eBioscience, and anti-Ly6G FITC (1A8) (Biolegend, SanDiego, CA, USA) and anti-CD19 PE (1D3) (BD Pharmingen, San Diego, CA, US). The cells were analyzed on a BD LSR-Fortessa™ flow cytometer (BD Biosciences, USA) and the data were further analyzed with Kaluza 2.1 software (Beckman Coulter, Inc., Brea CA, USA).

## 3. Results

### 3.1. CRAMP Expression in Neonatal Mice Is Reduced during C. parvum Infection

**Figure 1:**
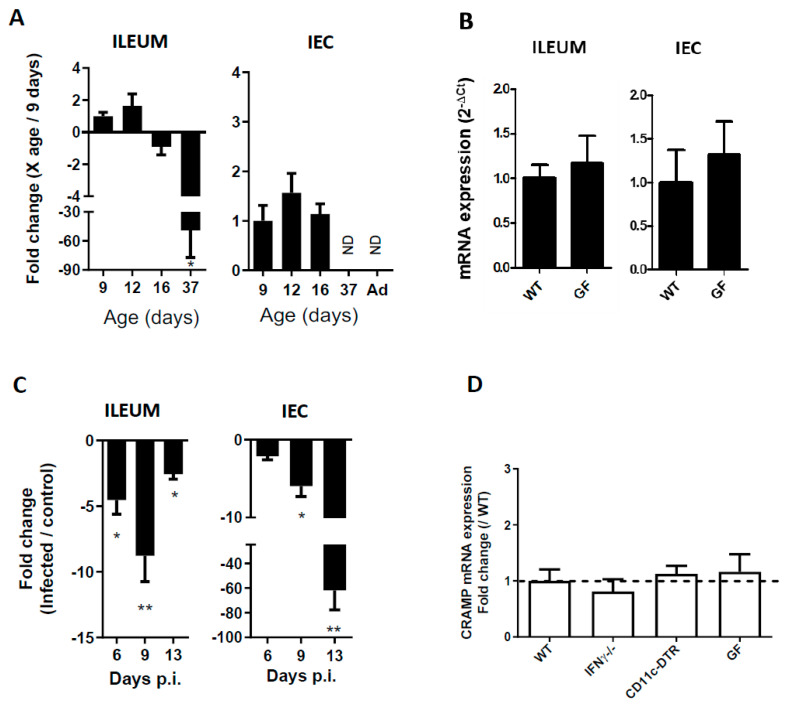
Ileal Cathelicidin Related Antimicrobial Peptide (CRAMP)expression is reduced during *Cryptosporidium parvum* infection of neonatal mice. mRNA expression is measured by qRT-PCR in different conditions. (**A**) CRAMP mRNA levels were determined in the whole mucosa of ileum (left panel) and in purified intestinal epithelial cells (IEC) (right panel) of non-infected mice at different ages. n ≥ 5 mice per group. (**B**) The absence of microbiota does not modify CRAMP expression. CRAMP mRNA levels were determined by qRT-PCR in the whole mucosa of ileum (left panel) and in purified intestinal epithelial cells (IEC) (right panel) of germ-free (GF) and Wild-Type (WT) neonatal mice at 16 days of age. (**C**) CRAMP mRNA expression is significantly decreased during *C. parvum* infection of neonatal mice. Three-day-old neonatal mice were orally infected with 5 × 10^5^ oocysts of *C. parvum* and euthanized at day 6, 9 and 13 pi for analysis of CRAMP expression in the ileum by qRT-PCR. Each group of mice is composed of 6 to 11 neonatal mice. (**D**) The reduced mRNA expression of CRAMP in the neonatal intestine is independent of the microbiota (GF mice) and IFNγ, and of CD11c+ cell recruitment. Three-day-old neonatal mice were orally infected with 5 × 10^5^ oocysts of *C. parvum* and euthanized at day 9 pi (for CD11cDTR and IFNγ^−/−^ mice) and 13 pi for GF mice. These data are from one experiment representative of two. Mann–Whitney non-parametric test * *p* < 0.05, ** *p* < 0.01. ND: not detected.

The intestinal antimicrobial response is different in neonates compared to adults [18]. We confirmed by qRT-PCR that CRAMP expression is significantly reduced in the ileum and in the intestinal epithelial cell (IEC) of 37-day-old mice (Figure 1A) compared to 9-day-old mice. A difference in microbiota composition may have affected CRAMP expression. We demonstrated in 16-day-old germ-free neonatal mice that in the absence of microbiota, CRAMP expression was not affected, ruling out this possibility (Figure 1B). We then analyzed the effect of *C. parvum* infection on intestinal CRAMP response in neonatal mice orally inoculated at three days of age. As previously described [20], the first oocysts were found in the intestine 4 days post-inoculation (dpi) and the peak of infection was reached at 9 dpi. Surprisingly, whereas CRAMP expression is often described to be strongly upregulated under inflammatory conditions [18,22,23], it was significantly downregulated upon infection by *C. parvum.* The stronger mRNA downregulation was observed in the intestine at day 9 dpi (Figure 1C left panel). In the intestine, CRAMP is constitutively expressed in the IECs of mice early after birth. As IEC are the only cells infected by *C. parvum*, we analyzed CRAMP mRNA in these cells by qRT-PCR. CRAMP expression was significantly reduced in infected neonates IECs’, demonstrating that these cells contribute to reducing CRAMP expression in the ileum (Figure 1C right panel).

### 3.2. The Reduced Expression of CRAMP Is Independent of IFNγ, Dendritic Cells and the Microbiota

Metabolites produced by microbiota such as butyrate are known modulators of Cathelicidin expression in the colonic epithelium [22,24]. We used germ-free neonatal mice to investigate the microbiota’s role on CRAMP expression during infection. After infection, the germ-free and conventional neonates presented similar parasitic loads (data not shown), and the decreased expression of CRAMP during infection was unaffected by the absence of microbiota (Figure 1D).

IFNγ, a key pro-inflammatory cytokine, is highly expressed during *C. parvum* infection, and is critical to the resolution of infection [20,25,26]. Moreover, this cytokine was shown to negatively modulate β-defensin1 during *C. parvum* infection [8]. To investigate the role of IFNγ in CRAMP expression, IFNγ-deficient neonates were infected with *C. parvum*. The reduction in CRAMP expression was similar to the one observed in Wild-Type mice (Figure 1D). We further searched for the direct or indirect role of CD11c positive cells in the modulation of CRAMP expression. These cells are recruited during infection [7,27] and can have beneficial or deleterious functions during infection. In infected neonatal mice depleted for CD11c cells that exhibited a higher parasitic load [7], there is no difference in CRAMP expression. Altogether, these results highlight that CRAMP downregulation seems to not be related to a specific feature of the neonatal immune system, or to inflammation induced during *C. parvum* infection.

### 3.3. Depletion of CRAMP in Neonatal Mice Does Not Worsen C. parvum Infection

**Figure 2:**
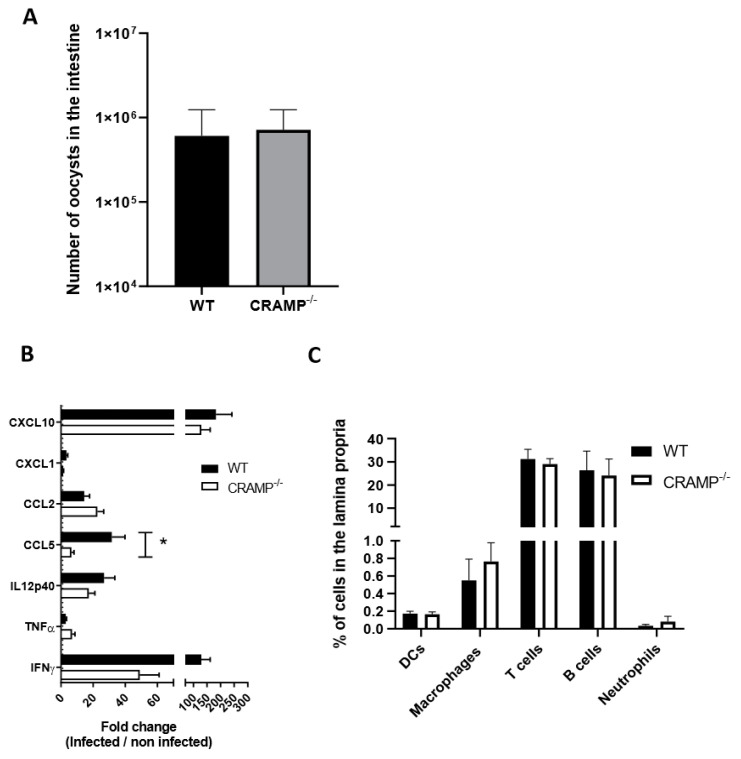
CRAMP deficiency did not increase the number of intestinal parasites. Three-day-old C57BL/6J and CRAMP^−/−^ neonates were orally infected with 5 × 10^5^ oocysts of *C. parvum*. (**A**) Parasitic load was evaluated in the intestines of neonatal mice after 5 days of infection. Mann–Whitney non-parametric test * *p* < 0.05. There were three repetitions of this experiment and the graph represents the cumulative data (n = 35). (**B**) qRT-PCR analyses of proinflammatory cytokine and chemokine expression in the ileum were performed in C57BL/6J and CRAMP^−/−^ neonates at day 5 pi (n = 6 mice per group). (**C**) The intestinal recruitment, induced by CRAMP, of dendritic cells, macrophages, T and B cells, and neutrophils was analyzed by flow cytometry in C57BL/6J and CRAMP^−/−^ neonates at day 5 pi (n = 6 mice per group). Mann–Whitney non-parametric test * *p* < 0.05. These data are from one experiment representative of two.

CRAMP plays a prominent role in the protection against infection of the newborn [18]. We therefore wondered if CRAMP can play a critical role in the control of *C. parvum* infection, and if its downregulation can be related to the higher parasitic load. CRAMP^−/−^ neonatal mice were inoculated at 3 days of age, and the number of parasites was counted at the beginning of infection at 5 dpi, when the level of ileal CRAMP expression is still elevated but begins to decrease. At this time point, no significant difference in the intestinal number of oocysts was observed between CRAMP^−/−^ and the WT neonatal mice (Figure 2A). This result suggests that the basal level of CRAMP is not sufficient to control *C. parvum* infection.

CRAMP is known to modulate a number of molecules associated with the immune response and inflammatory cytokine. We analyzed the mRNA expressions of selected inflammatory genes and observed a significantly higher expression of CCL5 after infection in WT neonatal mice compared to CRAMP^−/−^ mice (Figure 2B). We also performed flow cytometry analyses on ileal-infiltrating immune cells, including dendritic cells and macrophages, which play an important role in the control of the acute phase of infection [7,28]. We did not observe any significant difference in the recruitment of immune cells between WT and CRAMP^−/−^ neonatal mice (Figure 2C).

### 3.4. Oral Administrations of Recombinant CRAMP to C. parvum Infected Neonatal Mice Significantly Reduce Parasite Load

**Figure 3:**
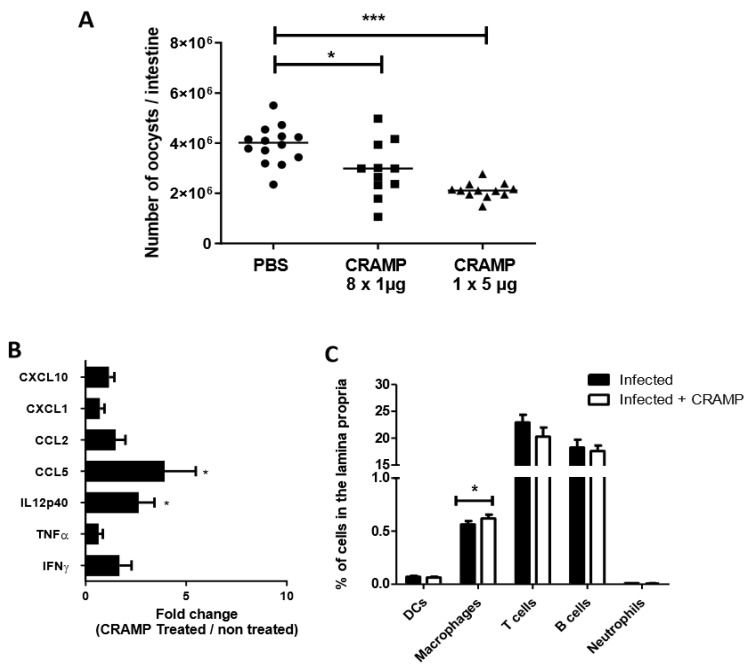
Oral administration of CRAMP inhibits *Cryptosporidium parvum* development in neonatal mice. (**A**) Three-day-old C57BL/6J neonates were orally infected with 5 × 10^5^ oocysts of *C. parvum*. CRAMP was administered orally each day (1 µg per day) for 8 days from the day of inoculation or only at day 8 pi (5 µg). Parasitic load was evaluated in the intestine of neonatal mice at 9 days of infection. (**B**) qRT-PCR analyses of proinflammatory cytokine and chemokine expression in the ileum were performed 4 h after the unique administration of 5 µg of CRAMP (n = 6 mice per group). (**C**) The intestinal recruitment induced by CRAMP of dendritic cells, macrophages, T and B cells and neutrophils was analyzed by flow cytometry 24 h after CRAMP administration. Mann–Whitney non-parametric test * *p* < 0.05, *** *p* < 0.0001. These data are from one experiment representative of two.

We next wondered if a higher quantity of CRAMP in the lumen of infected neonates may help to control parasite development. The protective effect of CRAMP on *C. parvum* infection was therefore investigated by therapy with exogenous CRAMP administration. In agreement with our hypothesis, the daily administration of recombinant CRAMP for 8 days from *C. parvum* inoculation significantly reduced the parasitic load in neonatal mice at 9 dpi (Figure 3A). Moreover, a single 5 µg oral administration of CRAMP to 5-dpi-infected neonates was sufficient to rapidly reduce oocyst number in the intestine (Figure 3A). As CRAMP displays immunomodulatory functions, we analyzed the ileal mRNA expression of various chemokines and inflammatory cytokines already described to be involved in the protective immune process of *C. parvum* infection. After oral administration of CRAMP, a significant increase in CCL5 and IL12p40 expression was observed in the intestines of treated neonatal mice. By flow cytometry analyses on ileal immune cells, we observed that CRAMP treatment resulted in increased amounts of infiltrating macrophages in the intestines of infected neonatal mice (Figure 3C). In contrast, CRAMP did not affect the percentage of dendritic cells, T cells, B cells or neutrophils infiltrating the intestine (Figure 3C). Taken together, these results suggest that the protection mediated by CRAMP induced significant modification of the chemokine CCL5, probably related to the higher rate macrophage recruitment observed. The significant difference observed in IL12p40 may also be associated with the higher number of macrophages.

### 3.5. The Stimulation of Enterocytes by CRAMP Does Not Change Their Permissiveness to C. parvum

**Figure 4:**
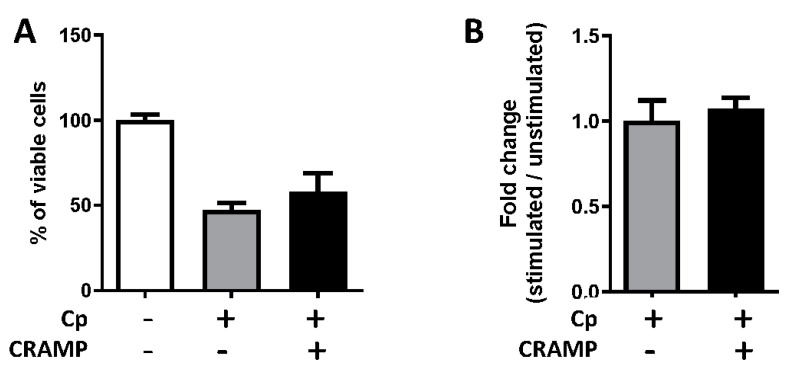
Stimulation of enterocytes by CRAMP does not modify their permissiveness to *C. parvum* infection. (**A**) mICcl2 cells were cultured to confluence and were stimulated or not with 100 ng/mL of CRAMP for 24 h, washed and infected or not by *Cryptosporidium parvum* (MOI 5) for an additional 24 h. Cell viability was analyzed with MTT test (**A**) and infection rates were analyzed by measuring RNA 18S expression by qRT-PCR (**B**). These results are representative of three independent experiments with similar results.

As effector host defense molecules, cathelicidins possess broad-spectrum antimicrobial and multifaceted immunomodulatory properties [29]. CRAMP can directly act in various cell types by activating pattern recognition receptors or affecting intracellular inflammatory signaling [30,31]. Moreover, CRAMP can prevent cellular apoptosis by modulating apoptotic pathways and associated proteins [32,33]. We therefore investigated the effect of CRAMP-stimulated intestinal epithelial cells on *C. parvum* infection. We did not observe any difference in the viability of infected epithelial cells after in vitro stimulation with CRAMP (Figure 4A), and did not observe difference in the level of infection (Figure 4B), suggesting that CRAMP does not reduce *C. parvum* oocyst numbers by acting on IECs.

### 3.6. CRAMP Displays Antimicrobial Activity against C. parvum Sporozoites

**Figure 5:**
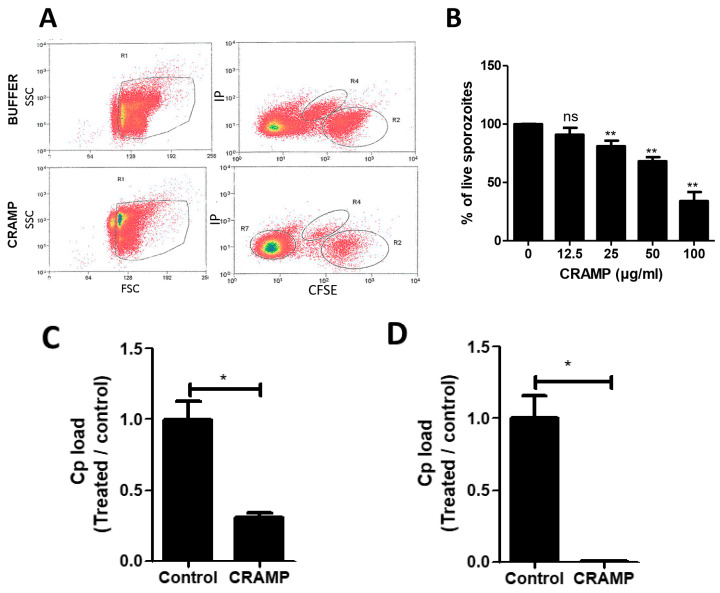
CRAMP displays antimicrobial activity against *C. parvum* sporozoites. (**A**) *C. parvum* sporozoites were purified and incubated with 50 µg/mL (**A**) or with different doses (**B**) of CRAMP for 3 h at 37 °C in phosphate buffer containing 50 mM NaCl. Sporozoites were incubated with CFDA-SE (8 µg/mL) and propidium iodide (5 µg/mL) to assess their viability by flow cytometry (**A**,**B**). Oocysts (**C**) and sporozoites (**D**) of *C. parvum* were incubated for 3 h with 100 µg/mL of CRAMP before their addition to mICcl2 cells (MOI 10). The infection rate was analyzed by quantifying *C. parvum* 18S RNA 24 h later by qRT-PCR. Statistical analyses * *p* < 0.05, ** *p* < 0.001.

To better understand how CRAMP can protect when orally administered to neonatal mice, we next investigated the antimicrobial effectivity of CRAMP on *C. parvum.* CRAMP displays antimicrobial activity against different bacteria, fungi, and also parasites such as *Leishmania* [34]. Three hours after exposure to CRAMP, viable sporozoites were stained with CFDA-SE and analyzed by flow cytometry. An important reduction in viable sporozoites was observed (Figure 5A), indicating that CRAMP exposure damages sporozoites in a dose-dependent manner (Figure 5B). Oocysts and sporozoites exposed to CRAMP significantly lost their ability to infect intestinal epithelial mICcl2 cells (Figure 5C,D). These data clearly show that CRAMP alters the viability of free sporozoites.

## 4. Discussion

Cathelicidins are small proteins of the innate immunity system, which display antimicrobial activities and constitute in living organisms a protective barrier against a number of potential pathogens, such as bacteria and viruses. The murine cathelicidin CRAMP is expressed by a variety of cells and tissues, and highly resembles the human cathelicidin (LL-37). Despite their important role described in fighting pathogens, they have been poorly investigated in parasitic infections. In vitro, LL37 was shown to contribute to the restriction of *Leishmania* development in THP1cells [35] and in primary human macrophages [34]. Moreover, *Plasmodium yoelii* and *Trypanosoma* viability and cellular multiplication are reduced when exposed to LL37 [36,37]. Several antimicrobial peptides of the cationic peptides and phospholipases families were shown to significantly reduce the viability of *C. parvum* sporozoites [9]. In addition, LL37 also leads to a reduction in sporozoite infectivity in vitro [19]. As an interspecies cathelicidin comparison revealed divergence in antimicrobial activity, TLR modulation, chemokine induction and the regulation of phagocytosis [38], we extended the result obtained with LL37 on *C. parvum*, and demonstrated that the murine cathelicidin CRAMP is also able to reduce the in vitro infectivity and viability of sporozoites at concentrations ranging from 25 to 100 µg/mL. In the small intestine, in response to infectious agents, cathelicidins are produced by many cell types such as epithelial cells, myeloid precursors, mast cells, etc. The unique study investigating the production of cathelicidins during *C. parvum* infection was performed in H69 monolayers by Hu et al. They highlighted the shuttling of both HBD2 and LL-37 in the apical exosomes released from *C. parvum*-infected H69 monolayers, and demonstrated their antimicrobial activity on *C. parvum* sporozoites [39]. This mechanism observed in vitro was suggested to contribute to mucosal anti-*C. parvum* defense.

The early life period—under 5 years for children and during the first month of the life for calves—is a critical period for sensitivity to *C. parvum*. During this particular period, diverse physiological and morphological changes are described in the intestine [40]. For example, the intestinal mucus layer is thinner, and the expression of antimicrobial peptides and their anatomical distribution are different from those of adult mice [18]. Similar changes in the composition of antimicrobial peptides and in bactericidal activity during the postnatal period in humans have been described [41]. Thus, CRAMP expression in the small intestine was shown to be elevated during the neonatal period. Associated with an increase in epithelial proliferation and enhanced Wnt signaling during the postnatal period, CRAMP expression was shown to be lost after 3 weeks of age. This higher CRAMP expression just after birth contributes to the protection from intestinal bacterial growth of the newborn enteric pathogen *Listeria monocytogenes* [18]. Surprisingly, whereas the expression of antimicrobial peptides such as cathelicidin is significantly increased during infections favoring the control of infection, such as listeriosis [18], our data demonstrated that the mRNA expression of CRAMP was significantly reduced in the small intestine of neonatal mice during *C. parvum* infection. We excluded the role of microbiota and of inflammatory responses with, IFNγ and dendritic cells as the main components in this downregulation. A direct ability of the parasite to evade the antimicrobial activity of CRAMP could explain this downregulation. To counteract host immunity, *C. parvum* has evolved multiple strategies to suppress host responses, such as β-Def1 [8] and CCL20 [10] response. Ming et al. demonstrated that downregulation of the DEFB1 gene was associated with host delivery of Cdg7_FLc_1000 RNA transcript, a *C. parvum* RNA that has been previously demonstrated to be delivered into the nuclei of infected host cells [42]. Other pathogens have developed strategies to escape the antimicrobial activity of CRAMP, such as *Entamoeba histolytica*, by producing protease to inactivate AMPs, and *Shigella flexneri* by a bacterial plasmid DNA [22,43]. More studies are needed to decipher the mechanism by which CRAMP downregulation occurs during *C. parvum* infection.

Despite the decreased CRAMP expression upon *C. parvum* infection, we wondered whether the elevated initial production of CRAMP during the neonatal period could favor the resistance of neonates, as described by Menard et al. for *Listeria monocytogenes* [18]. Surprisingly, our data did not reveal a significant effect of CRAMP deficiency on the intestinal parasite charge. This result did not rule out the possibility that exogenous administration of CRAMP could play a role in controlling parasite development. Thus, we administered CRAMP to neonatal mice and observed the control of parasite multiplication. This protection may be due to a direct effect of CRAMP on the parasite, as demonstrated by the loss of viability of sporozoites in the in vitro assays, but also by an indirect effect on immune cells. Indeed, beyond its direct antimicrobial activity, CRAMP or LL-37 have also been reported to modulate cytokine and chemokine production, apoptosis, functional angiogenesis or wound repair by stimulating keratinocyte migration and proliferation [44]. They can exert direct chemotactic actions on neutrophils, macrophages, immature dendritic cells, mast cells, monocytes and T lymphocytes [45]. It has been reported that LL37 can directly induce macrophages to differentiate into M1 types with proinflammatory effects, such as iNOS, CXCL9 and CXCL10 production [46]. Our data demonstrated that the protection by exogenous administration of CRAMP was associated with a moderate increased expression of CCL5 and IL12p40, and a significant increase in macrophage percentage. Altogether, these results can suggest that in addition to a direct effect of CRAMP on the parasite, protection induced by CRAMP could be mediated by the recruitment and activation of macrophages.

In conclusion, we described a putative new mechanism of host defense evasion by *C. parvum*, with an unexpected downregulation of the murine cathelicidin CRAMP during *C. parvum* infection of neonatal mice that could favor parasite multiplication. Further, our results provide news insights into the role of cathelicidins in controlling *Cryptosporidium parvum*. This reinforces the idea of using AMPs as a new non-specific method to control cryptosporidiosis. This could also constitute a potential alternative to conventional antibiotics avoiding antimicrobial resitance, which is a world health issue.

## Figures and Tables

**Table 1:** Primer Sequences.

Gene	Forward Primer Sequences	Reverse Primer Sequences
CCL2	5′-TGCTACTCATTCACCAGCAAGAT-3′	5′-GTGGTTGTGGAAAAGGTAGTGG-3′
CCL5	5′-TCTCTGCAGCTGCCCTCACC-3′	5′-TCTTGAACCCACTTCTTCTC-3′
CRAMP	5′-CCCAAGTCTGTGAGGTTCCG-3′	5′-AGGCAGGCCTACTACTCTGG-3′
CXCL1	5′-CGCTCGCTTCTCTGTGCAGC-3′	5′-GTGGCTATGACTTCGGTTTGG-3′
CXCL10	5′-CACGTGTTGAGATCATTGCCA-3′	5′-GCGTGGCTTCACTCCAGTTA-3′
IFNγ	5′-TCTTCTTGGATATCTGGAGGAA-3′	5′-AGCTCATTGAATGCTTGGCGCTG-3′
IL-12p40	5′-CTCACATCTGCTGCTCCACAA-3′	5′-GACGCCATTCCACATGTCACT-3′
PPIA	5′-GTCTCCTTCGAGCTGTTTGC-3′	5′-GATGCCAGGACCTGTATGCT-3′
TBP	5′-CAGCCTTCCACCTTATGCTC-3′	5′-TTGCTGCTGCTGTCTTTGTT-3′
TNFα	5′-ATGAGCACAGAAAGCATGATC-3′	5′-TACAGCCTTGTCACTCGAATT-3′
Cp18S	5′-CCGATAACGAACGAGACTCTGG-3′	5′-TAGAGATTGGAGGTTGTTCCT-3′
